# Discovering Vegetation Recovery and Landslide Activities in the Wenchuan Earthquake Area with Landsat Imagery

**DOI:** 10.3390/s21155243

**Published:** 2021-08-03

**Authors:** Cheng Zhong, Chang Li, Peng Gao, Hui Li

**Affiliations:** 1 Three Gorges Research Center for Geo-hazard, Ministry of Education, China University of Geosciences, Wuhan 430074, China; zhonglxm@cug.edu.cn (C.Z.); lichang_net@cug.edu.cn (C.L.); 2 Department of Earth and Ocean Sciences, University of North Carolina, Wilmington, NC 28403, USA; gaop@uncw.edu; 3 Department of Geography, University of South Carolina, Columbia, SC 29208, USA; 4 School of Earth Sciences, China University of Geosciences, Wuhan 430074, China

**Keywords:** earthquake, landslide, vegetation recovery, *NDVI*, slope stability, Landsat, land cover

## Abstract

Post-seismic vegetation recovery is critical to local ecosystem recovery and slope stability, especially in the Wenchuan earthquake area where tens of thousands of landslides were triggered. This study executed a decadal monitoring of post-seismic landslide activities all over the region by investigating landslide vegetation recovery rate (*VRR*) with Landsat images and a (nearly) complete landslide inventory. Thirty thousand landslides that were larger than nine pixels were chosen for *VRR* analysis, to reduce the influence of mixed pixels and support detailed investigation within landslides. The study indicates that about 60% of landslide vegetation gets close to the pre-earthquake level in ten years and is expected to recover to the pre-earthquake level within 20 years. The vegetation recovery is significantly influenced by topographic factors, especially elevation and slope, while it is barely related to the distance to epicenter, fault ruptures, and rivers. This study checked and improved the knowledge of vegetation recovery and landslide stability in the area, based on a detailed investigation.

## 1. Introduction

A strong earthquake often causes thousands of landslides, debris flows, and other secondary geohazards, which affects slopes’ stability for a long time [1]. Those landslides or instable slopes are easily re-activated in the following rainy seasons [2,3,4]. At 14:28 on 12 May 2008, a Ms 8.0 earthquake occurred in Wenchuan County, Sichuan Province. As of 8 October 2009, this earthquake saw 69,229 people killed, 17,923 people missing, and 374,643 injured [5,6,7,8]. Tens of thousands of landslides were triggered by the earthquake and caused tremendous property loss and casualties [9,10,11]. The post-seismic landslide event has attracted extensive attention, as the number, density, area, and casualties of landslides in the event are all much greater than those in other events caused by a single earthquake [12,13]. Several studies forecasted that post-seismic landslide activities will continue for 20 years or more [14,15].

The status of vegetation recovery after geological disasters is an important reference for evaluating local ecosystem recovery and slope stability [16,17,18]. The vegetation canopy absorbs a part of the rainfall and decrease water infiltration to the soil and, thereby, prevents the sudden increase of soil moisture content [19,20]. Vegetation roots extract the soil moisture and increase matric suction in the unsaturated root zone, which leads to more stabilization [21]. Plants can effectively improve the resistance to impacts and shear strength of a slope by reinforcing and anchoring slope soil with their root systems [22]. In landslide areas, plants produce more below-ground biomass as a morphological adaptation to acquire more water and nutrients [23]. Hence, vegetation is wildly used as a promising sustainable biotechnical engineering for slope stabilization [24].

Monitoring vegetation status with remote sensing images is believed to be an effective alternative to a regional investigation, in comparison with field surveys [25]. In the Wenchuan earthquake area, serval related studies have been reported. The literature [26] analyzed the spatial and temporal patterns of post-seismic landslide changes by monitoring the vegetation recovery status with images. A study [27] also investigated the influences of precipitation and topography on vegetation recovery in the Wenchuan earthquake area. A decadal analysis of vegetation recovery and landslide activity was conducted in the literature [28] and found that about 83% of vegetation has recovered. In these studies, MODIS products (e.g., *NDVI*) are often used as the main data sources, leading to great uncertainties in estimating the vegetation recovery. In the MODIS image, most “landslide pixels” are actually a mixture of landslide and other covers (e.g., vegetation), as 99.25% of post-seismic landslides are smaller than a MODIS pixel (250 m × 250 m). In studies with MODIS images, the vegetation damage or vegetation recovery rate of most landslide pixels would deviate from the true status, as non-landslide surfaces are involved. This serious error may result in incorrect decisions or policies for eco-environment protection and landslide prevention.

In order to improve the knowledge of decadal vegetation recovery and landslide stability in the Wenchuan Earthquake area, this study conducted a detailed and complete monitoring of the vegetation recovery status of post-seismic landslides with Landsat images, given its image pixel is less than 83.56% of those landslides.

## 2. Materials and Methods

### 2.1. The Study Area

**Figure 1:**
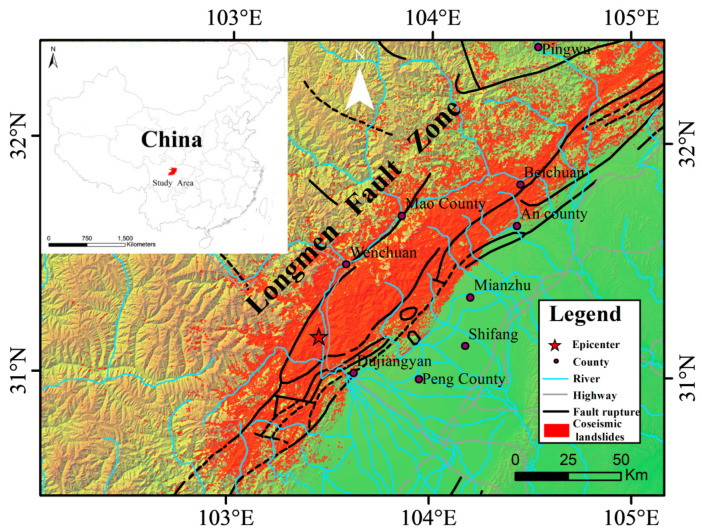
Study area map showing landslides triggered by the 2008 Wenchuan earthquake, fault rupture, location of Wenchuan, Beichuan, etc.

As shown in Figure 1, most post-seismic landslides happened within the Longmenshan fault zone, the junction of the front edge of the Qinghai–Tibet Plateau and the Sichuan Basin. The area is characterized by mountainous landscapes (e.g., high mountains and deep valleys), which were created by strong neotectonic movement. The Longmenshan fault zone consists of several fault ruptures including the Maowen fault, Yingxiu–Beichuan fault, and the Dujiangyan–Anxian fault [29], where earthquakes or geohazards may happen at some time. The area has a temperate monsoon climate. The annual mean sunshine duration, temperature, and precipitation are 1600 h, 12.9 °C, and 719.7 mm, respectively. Most rainfall happens in the growing season (May to September), which probably raises the risk of landslides, debris flows, and other disasters. In the area, eight vertical natural climate zones are identified along with the altitude rising from southeast to northwest. More than 60% of this area had moderate or high vegetation coverage before the known earthquake.

In all, 197,481 secondary landslides were triggered by the earthquake, and their total area was about 1160 km^2^ [30]. These landslides were distributed in a great region (about 110,000 km^2^) in the Longmenshan fault zone, with elevation from 800 to 4500 m. Most landslides are characterized by high elevation, large volume, low compactness, and heterogeneous components [8,9]. They are easily re-activated and effected by the heavy and unevenly distributed precipitation in this area. After the earthquake, the affected areas exhibited landslides, debris flows, and remobilizations in the subsequent monsoon periods [12,31]. In this area, vegetation has been seriously destroyed by the earthquake and following geological disasters (e.g., landslides, debris flows, and collapses) [8,16]. The recovery of vegetation and the local ecosystem is considered a difficult and long-term process [17] as the organic matter on the surface has probably been completely removed due to the slope movement and because the surface may often be washed or even pushed away by heavy rainfall.

### 2.2. Data

#### 2.2.1. Landsat Images

Remote sensing has been widely used in vegetation mapping and landslide monitoring, as it can capture plentiful spectral information at regional and global scales quickly and periodically [32]. Landsat images were used to monitor vegetation recovery in this study, as they have moderate resolution (30 m) and free long-term archives (since the 1970s) (https://earthexplorer.usgs.gov/, accessed on 1 August 2021). In order to cover the study area, four scenes (e.g., track no. 130/38, 129/38, 130/39, and 129/37) were needed. Given that May to September is the vegetation growth season of this area, all Landsat images (e.g., TM, ETM+, OLI, and TIRS), and Landsat-like images (e.g., HJ-1 (http://www.rscloudmart.com/, accessed on 1 August 2021), Sentinel-2 (https://scihub.copernicus.eu/dhus/#/home, accessed on 1 August 2021)) in this period of the years 2007 to 2018 were involved and fused, to reduce contamination due to clouds, haze, snow, or ice. Among them, the ETM+ SLC-OFF images of 2012 were repaired with the triangulation method, as Landsat TM and OLI images were unavailable in that year.

#### 2.2.2. Other Data

The landslide polygons used in this study were interactively interpreted from high-resolution images (SPOT, QuickBird, etc.) after the earthquake [30]. The location and boundary of each landslide were delineated by computer-screen-based visual interpretation of images covering the whole area, including 23 post-earthquake images (consisting of aerial photographs, SPOT 5, CBERS02B, IKONOS, ASTER, IRS-P5, QuickBird, and ALOS) and 63 pre-earthquake images (consisting of SPOT 5 and ETM+). It is believed to be the largest and most complete post-seismic landslide inventory for the Wenchuan earthquake. In order to reduce the influence of mixed pixels or the matching error between the images and polygon data, 33,123 landslides bigger than nine Landsat pixels were chosen for vegetation recovery estimation, though 83.56% of the 197,481 landslides were greater than a Landsat pixel. In order to show landslides and their vegetation coverage clearly, 2781 landslides that were larger than 50 pixels were displayed in related figures.

ASTER GDEM (30 m) and MODIS *NDVI* (250 m) were downloaded from the Geospatial Data Cloud (http://www.gscloud.cn/, accessed on 1 August 2021). The former was used to evaluate the influence of landform factors (e.g., elevation, slope, and aspect) on vegetation recovery, while the latter were used as a comparison with Landsat images.

Geological map, earthquake data (epicenter, aftershocks, and affected areas, etc.), topographic maps, and meteorological and socioeconomic data were obtained from the related departments of the Sichuan government.

### 2.3. Methods

**Figure 2:**
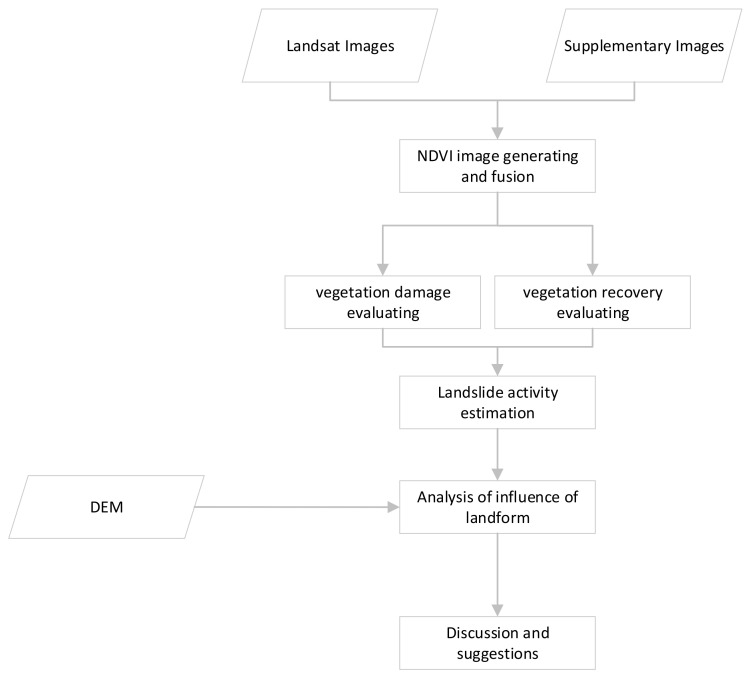
A Flowchart of this study.

The flowchart of this study is shown in Figure 2. First, *NDVI* images from the growing season of each year were generated and fused using the MVC method; secondly, the vegetation damage after the earthquake was estimated by comparing the *NDVI* images before and after the earthquake; thirdly, the vegetation recovery status was evaluated by calculating the difference between the *NDVI* right after the earthquake and the *NDVI* of a following year; then, a landslide’s activity was estimated roughly according to its vegetation recovery status; fifthly, the influence of the landform factors on vegetation recovery and landslide activity was analyzed; at last, discussion and suggestions for regional landslide stability and mitigation were given.

#### 2.3.1. *NDVI* Images Generating and Fusion

The normalized difference vegetation index (*NDVI*) is the most successful and widely applied index among many vegetation indexes. *NDVI* can effectively eliminate the interference of solar altitude angle, satellite scanning angle, and atmospheric conditions, by using the ratio between sensor channels. Compared with other indexes, such as the enhanced vegetation index (EVI), *NDVI* could depict plant growth, coverage, biomass, etc., more accurately, especially in low-vegetated areas. In this study, *NDVI* was used to evaluate vegetation damage and recovery status as previous researchers did, given the vegetation (forest or biomass) was seriously destroyed on the post-seismic landslide surface. *NDVI* can be obtained according to the following formula:

(1) $$NDVI=\frac{NIR-RED}{NIR+RED}$$

where *RED* and *NIR* are band 3 and band 4 in Landsat TM or ETM + reflectance, or band 4 and band 5 in Landsat OLI. For each year, all Landsat images in the growing season were used to produce *NDVI* images. Then, all *NDVI* images were fused with the maximum-value composite (MVC) method, to generate a cloud-free image. It is expected that this fusion could effectively reduce the contamination due to cloud, haze, ice, snow, or shadow. At last, the fused *NDVI* image was carefully checked by experts to find and remove contaminated pixels within landslides.

#### 2.3.2. Evaluating Vegetation Damage

In order to estimate the status of vegetation recovery accurately, the vegetation damage (*VDA*) caused by the Wenchuan earthquake should be investigated first, according to the following formula:

(2) $$VDA=\frac{NDVI_{0}-NDVI_{d}}{NDVI_{0}}\times 100\%$$

where $NDVI_{0}$ is the fused *NDVI* image of 2007, $NDVI_{d}$ indicates the *NDVI* image after the earthquake in 2008. For each pixel, VDA > 0 means the *NDVI* value after the earthquake is less than that before the event, which suggests that vegetation at the pixel was damaged by the earthquake; VDA= 0 suggests there is no vegetation damage; while VDA<0 implies vegetations coverage increases at this pixel. Generally, vegetation loss is an apparent indicator for interpreting landslides from high-resolution images visually. However, mixed pixels, noises, or matching errors between polygons and image may bring some errors.

As most landslides are greater than one Landsat pixel, their inner vegetation damage could be detected and mapped with the pixel-based *VDA* calculation. It probably happens that the vegetation in some pixels is seriously destroyed, while that in other pixels is barely damaged. Finer *VDA* and vegetation recovery evaluation could help develop more rational and skillful strategies.

#### 2.3.3. Evaluating Vegetation Recovery

In a year after the earthquake, the vegetation recovery status could be considered as the difference between *NDVI* of that year and *NDVI* right after the earthquake. In order to compare vegetation recovery status among years, the vegetation damage $NDVI_{0}-NDVI_{d\;}$is adopted as the denominator, and then a normalized formula, named the vegetation recovery rate (*VRR*), is presented as follows:

(3) $$VRR=\frac{NDVI_{r}-NDVI_{d}}{NDVI_{0}-NDVI_{d}}\times 100\%$$

where$\;NDVI_{r}$ is the fused *NDVI* image of a year after the earthquake, $NDVI_{d}$ and $NDVI_{0}\;$are the same as those introduced in Equation (2). In order to show the vegetation recovery status more clearly, the VRR values are often divided into six categories and as shown in Table 1.

Vegetation is often considered as an essential indicator of slope stability [19]. Here, we also use VRR to evaluate landslide activity as previous studies did [21]. The VRR values and categories and corresponding landslide activities are shown in Table 1. Specifically, landslide pixels with poor vegetation recovery (VRR Type I to Type III) are classified as active slopes; pixels with good vegetation recovery (VRR Type IV to Type V) are termed as weak active slopes; and pixels with excellent vegetation recovery (VRR Type VI) are thought to be inactive slopes. For a landslide, if more than one half pixels belong to active slopes, it is termed as an active landslide.

#### 2.3.4. The Influence of Landform

Vegetation growth is mainly controlled by temperature, water, and sunlight duration [33,34,35]. In the high mountainous area, landform factors, e.g., elevation, slope, and aspect, often have significant impacts on microclimate conditions for vegetation. For instance, vegetation in low valleys usually grows more quickly than that on high mountains (>5500 m) due to higher temperature, more water, and less cloud and snow. Thus, analyzing the influence of landform factors could help to reveal and understand the spatial–temporal patterns of vegetation recovery. In order to display the analysis clearly, elevation, slope, and aspect derived from the 30 m DEM, are divided into eight classes, as shown in Table 2.

## 3. Results

### 3.1. Vegetation Damage Evaluation

**Figure 3:**
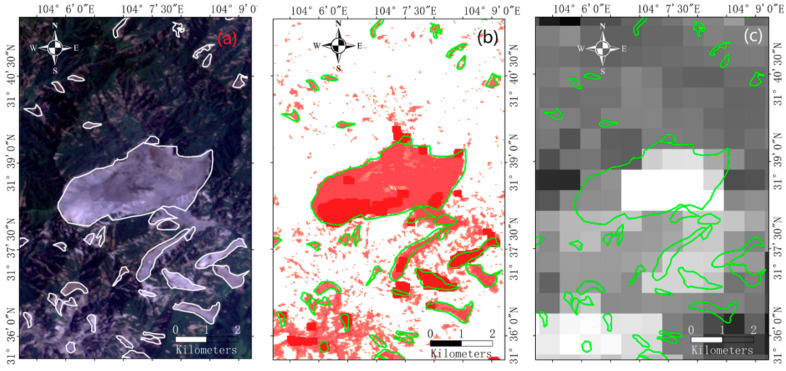

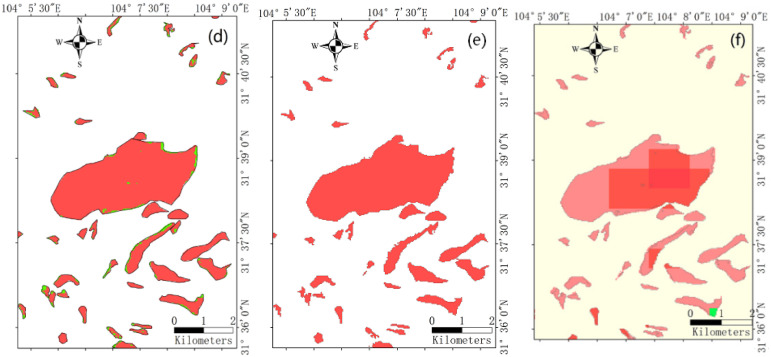
Examples of *VDA* analysis: (**a**) landslide polygons and the Landsat TM image in 2008; (**b**) landslide polygons and the *VDA* map (the bright red indicates the greatest *VDA* level); (**c**) landslide polygons and the MODIS NDVI image in 2008; (**d**) *VDA* of selected landslides, where *VDA* FALSE is in green; (**e**) landslide pixels, where *VDA* FALSE areas in panel (**d**) are removed; (**f**) the *VDA* map obtained from MODIS NDVI. All displayed landslides are greater than 50 Landsat pixels, and the greatest known one is the Daguangbao landslide (7.12 km^2^).

An example of the whole vegetation damage map (see Figure S1) was shown in Figure 3. In Figure 3b,d, most landslide pixels in red had TRUE *VDA*, only a few pixels got FALSE *VDA* (in green in Figure 3d). Errors around landslides boundary were probably caused by mixed pixels or matching errors between landslide polygons and the image. Errors in landslide might come from image noises. Both errors were carefully checked with original Landsat images (Figure 3a) and removed (as shown in Figure 3e). In Figure 3b, the *VDA* level of each landslide pixel was displayed, while most landslides were less than one pixel of MODIS *NDVI* image (shown in Figure 3c) and detailed vegetation damage could not be found from the corresponding *VDA* map (shown in Figure 3f).

### 3.2. Vegetation Recovery Rate

**Figure 4:**
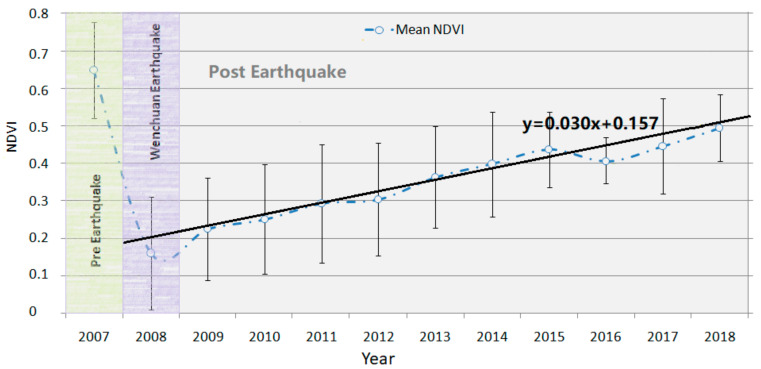
Mean NDVI (blue scatter plot with error bar indicating the root mean square error) of landslide pixels between the years 2007 and 2018.

As shown in Figure 4, the mean *NDVI* of landslide pixels was about 0.65 before the earthquake, and then sharply declined to 0.17 after the 2008 earthquake. From 2009 to 2018, the *NDVI* slowly increased with a clear trend (0.03/year) and approached 0.5 at the end. This general trend is in accord with previous studies in the earthquake affected area based on Landsat image [26], though their study region and time may be a little different. It should be pointed out that about 16.57% of landslide pixels were removed as they were covered by clouds, snow, or haze and could not be repaired by fusing all *NDVI* images in one year.

According to Table 3, the percentage of pixels falling in *VRR* Type I to Type III decreased from 97.3% in the year 2009 to less than 10% within 10 years. It is noticeable that pixels in Type I were just 7.7% in 2009 and then decreased to 0, which implies the vegetation of most pixels began to recover right after the earthquake. The *VRR* type with the most falling pixels was Type II from 2009 to 2010, Type III from 2011 to 2014, Type IV from 2015 to 2017, and Type V in 2018, which indicated that most vegetation has gradually recovered since 2009. As no vegetation has re-grown better than the pre-earthquake level by 2018, there is no Type VI in Table 3.

**Figure 5:**
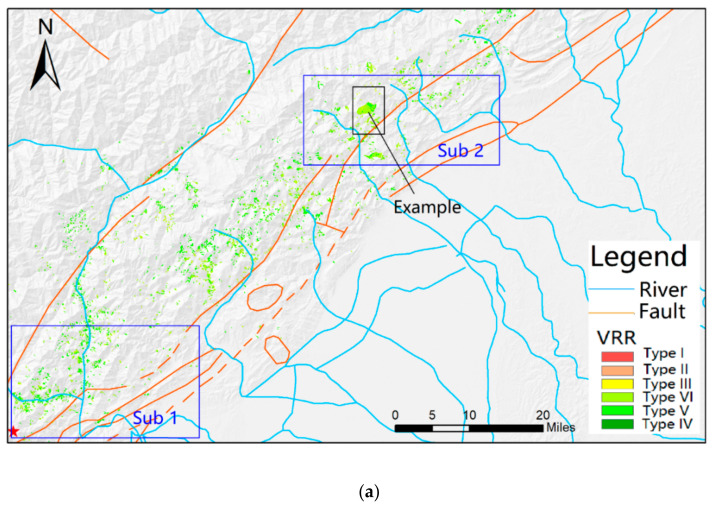

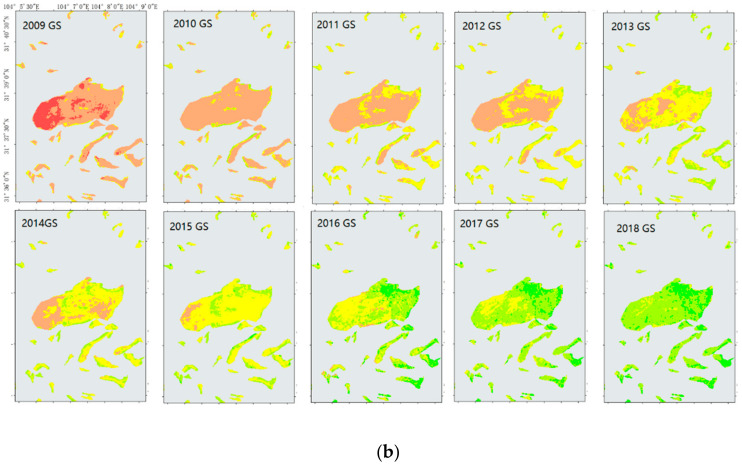
The VRR map. (**a**) the overall *VRR* map of 2018, and (**b**) an example of vegetation recovery process during the study years. Here, GS stands for growing season. The sub-regions in the *VRR* map are used for analyzing the influences of the epicenter, fault ruptures, and rivers, as shown in Figure 7.

Figure 5 shows the overall vegetation recovery map of 2018. According to the map, vegetations of most landslides were well recovered (*VRR* is Type IV or Type V) in 2018. It is seen that the spatial distribution of *VRR* was not related to the distance from the landslides to the epicenter or fault ruptures. Landslides located in the Longmenshan Fault Zone (between the Maowen Fault and the Yingxiu–Beichuan Fault) and those outside the fault zone, had similar *VRRs*.

The yearly *VRR* maps of a subregion around the Daguangbao landslide is taken as an example to show spatial–temporal patterns and details of landslide vegetation recovery. In general, vegetation recovery patterns of this area are in accord with that shown in Table 3, with a remarkable fact that the *VRR* of the Daguangbao landslide was apparently lower than that of surrounding landslides in each year. It indicates the vegetation of the greatest landslide was serious damaged and hard to recover. Detailed investigation on the Daguangbao landslide revealed that eastern part had the largest *VRR* followed by the central part and west part, which can be explained by several reasons. First, the western part was the main scarp of the landslide [5], whose vegetation and soil were seriously destroyed by the earthquake and secondary landslides. Second, this part was much higher than other parts (elevation difference between the west and east is greater than 1500 m). Then, the climatic conditions for vegetation growth, e.g., temperature, sunlight duration, and water in this part, were much worse, which also limited vegetation recovery. For other smaller landslides, inner spatial variability of vegetation re-growth was also witnessed.

### 3.3. Landslide Activity Estimation

Activities of post-seismic landslides were estimated based on their vegetation recovery status as shown in Table 4. According to Table 4, the percentage of active landslide gradually decreased within the decade, from 100% in 2009 to 5.88% in 2018; while the percentage of weak active landslide gradually increased during the study periods, from 0.0% in 2009 to 94.12% in 2018. It is noticed that the percentage of active landslides barely reduced during the first three years when vegetation was at the first stage of recovery. During these years, post-seismic landslides or unstable slopes were still easily re-activated. At the end of the study period, most landslides achieved a relatively stable status, though they did not reach the pre-earthquake level yet.

### 3.4. Influences of Landform Factors

**Figure 6:**
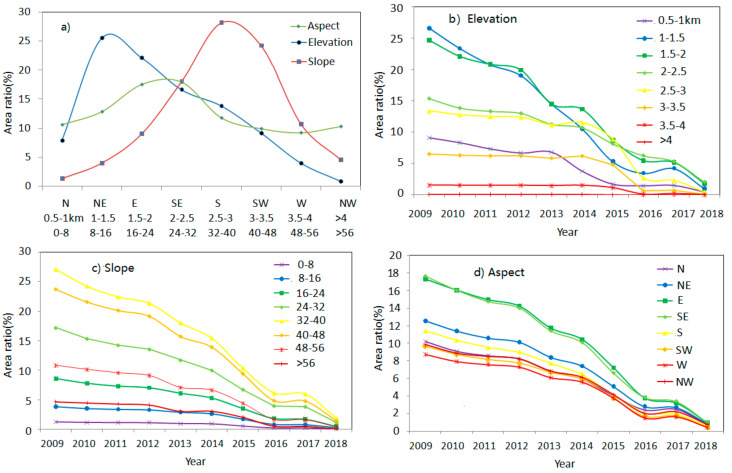
The influences of landform factors on active landslides. (**a**) Post-seismic landslides at different landforms; (**b**) active landslides at different elevation, (**c**) aspect, and (**d**) slope.

According to Figure 6a, landslides are mainly observed to occur at elevations from1000 to 2000 m, on slopes from 32° to 48°, and in the eastern and south-eastern slope directions, which is in agreement with previous research [15,30]. The area percentage of active landslide clearly decreased at all elevations, slopes, and aspects from 2009 to 2018, with remarkable spatial variability (as shown in Figure 6b–d).

In Figure 6b, the area percentage of active landslide at elevations from 1000 to 3000 m decreased much quicker than that at others. At these elevations, vegetation could recover better, due to higher temperature, more sunlight duration, and water. Landslides at elevations from 500 to 1000 m were probably in deep valleys, where sunlight is insufficient. At high mountains (>3000 m), vegetation recovery was limited by low temperature, serious clouds and snow, and soil and water losses. In Figure 6c, the area percentage of active landslide at moderate slope (24°–48°) reduced faster than that at others. At steeper slopes (>48°), surfaces covered by exposed bedrock or a little soil, were not suitable for vegetation growth [11,27]. At gentle slopes (<24°), landslides were not easily triggered; thus, their percentage was small, and the change was not evident. In Figure 6d, the area percentage of active landslide at slope directions E and SE reduced more quickly, while those in other aspects had a similar decrease rate. This may be explained by the fact that vegetation in these aspects was more benefitted by the SE monsoonal system.

### 3.5. Influences of Epicenter, Fault Ruptures, and Rivers

**Figure 7:**
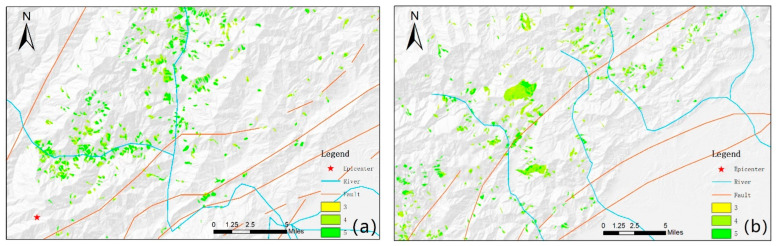
The partial *VRR* map of 2018 with the epicenter, faults, and rivers; (**a**) target area 1; and (**b**) target area 2. The blurred patch at the southwest of Figure 6c is a defect in the original DEM.

Figure 5 shows the spatial distribution of *VRR* was not related to the distance of landslides to the epicenter or fault ruptures. Subregions (e.g., Sub 1 and Sub 2) showed similar *VRR* distribution, implying that vegetation possessed similar growth condition (e.g., temperature, precipitation, and landforms, etc.) at a regional scale. In target area 1 (Figure 7a), landslides along rivers had a higher *VRR* level; while in target area 2 (Figure 7b), that phenomenon was not observed. This suggests that there is no evident relationship between *VRR* distribution and the distance to rivers.

## 4. Discussions

### 4.1. Characteristics of This Study

In this study, 197,481 landslides triggered by the 2008 Wenchuan earthquake were distributed in a 110,000 km^2^ mountainous regions where field surveys are very hard to execute. Previous studies have found and demonstrated that time series of *NDVI* are useful for monitoring the process of vegetation recovery and then evaluating regional geological stability after a large disturbance, by obtaining long-term and intensive observations of vegetation dynamics [11,27]. Moreover, time series data can help eliminate the contaminations of cloud, snow, and other noises.

The MODIS *NDVI* product has been widely used in monitoring vegetation recovery and geological stability [28], as it has high temporal resolution and is freely accessible. However, 99.25% of landslides triggered by the earthquake are smaller than a MODIS pixel; thus, most “landslide” pixels in related studies are actually a mixture of landslide and other land cover (e.g., vegetation). Analysis based on those pixels may be inaccurate and unreliable to some extent. What is worse, using these data cannot reveal the spatial variability within most landslides.

In order to check and improve the knowledge on vegetation recovery and landslide stability in the area, a decadal monitoring of vegetation recovery is executed based on fused Landsat images in the growing season of each year in this study. In general, our results are similar to that of previous researchers in this area. For instance, the trend of *NDVI* increasing is accord with that in literature [26], while being greater than that seen in the literature [28]. Furthermore, the spatial distributions of post-seismic landslide *VDA* and *VRR* are similar to those in literature ([26] and [30], respectively). It is found that topographic factors rather than the epicenter, fault ruptures, or rivers have significant influences on vegetation recovery as discovered in literature [28]. Those similarities demonstrate the validity and reliability of this study.

Ideally, more than 165,000 landslides that are bigger than a Landsat pixel can be investigated with the proposed method. To reduce the influence of mixed pixels, 33,123 landslides bigger than nine Landsat pixels, were selected for *VDA* and *VRR* analysis. The number of landslides involved this study is much greater than that (several thousands) in previous investigations. In addition, for all selected landslides, investigations on inner pixels could reveal more detailed vegetation recovery processes and slope activities, which is always difficult for field investigations in high mountainous area or studies with coarse-resolution images.

### 4.2. Uncertainty Analysis

In the process of evaluating *VDA*, errors around landslide boundaries were found, which were probably caused by mixed pixels and matching error between polygons and images. Other isolated errors in landslide polygons may result from image noises. The former error was directly removed without repairing, while the latter error was repaired with surrounding pixels, as it was often very small compared with a landslide. Overall, both errors just accounted for less than 0.5% of landslide pixels and barely affected the investigation and results. Overall, these errors may cause serious consequences in MODIS images, i.e., the number of landslides involved for further analysis may evidently decrease [28], as many landslides may be smaller than a single noise pixel and thus need to be removed.

In this high mountain area, images are often seriously contaminated by cloud, haze, snow, or ice, no matter what the type of image (e.g., MODIS, Landsat, or Sentinel). The contaminated area could be detected by checking the *NDVI* value or using the Fmask algorithm [36]. In this study, fusing all *NDVI* images in the growing season is expected to repair those contaminated pixels, though 16.57% pixels were contaminated in all images. However, it is hard to determine whether a pixel is influenced by light haze or not, especially in MODIS images, where light haze or even thick cloud may be involved in mixed pixels. Fusing *NDVI* images and times series analysis is expected to reduce this influence to some extent though it is difficult to evaluate.

Gaps in Landsat ETM+ images may bring uncertainties to our analysis, especially in 2012 when Landsat TM and OLI were unavailable. In other years, ETM+ images were just used as supplements, and they could not influence *VRR* analysis much. Although the ETM+ images of 2012 were repaired with the triangulation method, it is believed that uncertainties still existed. Fortunately, images of most years were not affected by the gaps, thus the general tendency of *VRR* is reliable.

### 4.3. Decay of Landslide Activity

Previous studies forecasted that post-event landslide activities will probably continue for about two decades [37]. It will be a long-term process for damaged vegetation to recover to the pre-earthquake level, as the earthquake-affected area is characterized by low precipitation, high evaporation, seasonal alterations, and dry and barren soil [38]. In this study, Landsat *NDVI* images were successfully used to investigate vegetation damage, recovery rate, and post-seismic landslide decay rate, in the high mountainous area with few human activities [26].

This work finds that about 60% of landslide vegetation recovered to higher than 75% of the pre-earthquake level after a decade, though no vegetation achieved or exceeded 100%. It is expected that vegetation can completely recover to pre-earthquake levels within 20 years, based on the current *NDVI* tendency and climate conditions. This predication agrees with those in previous studies [27,31], though the data, scale, methods, or study areas in them were different from ours. This study finds that the recovery rate of landslide vegetation is not related to its distance to the epicenter, fault ruptures, or rivers. Topographic factors, especially the elevation and slope, have a significant influence on the vegetation recovery rate, and they affect landslide activities indirectly [39]. Thus, these factors must be considered when one evaluates the stability of a region or just a single landslide in this area. Although climate factors did not cause evident *VRR* spatial variability in different subregions, they are still the essential factors for vegetation growth.

More importantly, extreme rainfall has been widely believed to be the most important factor activating post-seismic landslides and debris flows [40,41]. After the 2008 earthquake, Wenchuan has witnessed thousands of reactivated landslides and debris flows in almost every rainy season [42]. Fortunately, vegetation recovery could effectively reduce post-seismic landslide activities, and the number of reactivated landslides has become much less since the year 2017 [43].

Some eco-friendly measures have been conducted to mitigate landslides around human settlements. First of all, the disturbance or damage to the ecological environment should be minimized in the process of landslide investigation and mitigation. Pit exploration, trenching, and large-scale excavation should not be used as far as possible. After the drilling exploration, holes should be sealed in time. The design and construction of landslide treatment should be optimized according to the actual construction conditions, to reduce the excavation and cutting quantities. Greening is an eco-friendly method of landslide mitigation. The plant layout, species selection, and plant community collocation should be carefully planned to enhance the eco-geological functions of artificial vegetation, such as consolidating soil, absorbing water, and beautifying the environment. Benefiting from the short-term human intervention, the ecological environment could gradually be restored with the help of its self-regulation and self-recovery ability.

## 5. Conclusions

Tens of thousands of landslides triggered by the Wenchuan earthquake are easily re-activated by heavy and uneven precipitation. Thus, long-term monitoring of the status of landslide activities is critical to local ecosystem recovery and regional stability. Given that previous studies have demonstrated the link between vegetation recovery rate and landslide activities, this study executed an independent decadal monitoring of the vegetation recovery rate and post-seismic landslide activities in this area with Landsat images and a complete landslide inventory. More than 30,000 landslides bigger than nine Landsat pixels were selected for *VDA* and *VRR* analysis, which could help to reduce the influence of mixed pixels and support detailed investigations within a landslide. The study discovers that 60% of landslide vegetation recovered to higher than 75% of the pre-earthquake level after a decade, and most vegetation can completely recover to the pre-earthquake level within 20 years. The vegetation recovery rate is significantly influenced by topographic factors, especially the elevation and slope, but not related to the distance to the epicenter, fault ruptures, or rivers. Climate factors did not cause evident *VRR* spatial variability, though they are essential factors.

This study checked and improved the knowledge of vegetation recovery and landslide stability in the area, based on more comprehensive and detailed investigations. The results are of great help for clarifying disagreements between previous studies and for developing suitable policies for eco-environment protection and landslide treatment.

## Figures and Tables

**Table 1:** *VRR* value, type, and the corresponding landslide activities.

*VRR* Value (%)	*VRR* Type	Landslide Activity
<0	I	Active
0–25	II	
25–50	III	
50–75	IV	Weak active
75–100	V	
>100	VI	Inactive

**Table 2:** Categories of landform factor.

Class	Elevation (m)	Slope (ᵒ)	Aspect
1	500–1000	0–8	North
2	1000–1500	8–16	North East
3	1500–2000	16–24	East
4	2000–2500	24–32	South East
5	2500–3000	32–40	South
6	3000–3500	40–48	South West
7	3500–4000	48–56	West
8	>4000	>56	North West

**Table 3:** Area (km^2^) and percentage for different *VRR* types between 2009 and 2018.

Year	Type I		Type II		Type III		Type IV		Type V	
	Area	%	Area	%	Area	%	Area	%	Area	%
2009	52.15	7.7	421.56	62.4	183.50	27.2	18.18	2.7	0	0
2010	0	0	367.01	54.3	229.84	34.0	77.63	11.5	0.92	0.1
2011	0	0	153.87	22.8	404.21	59.8	117.32	17.4	0.09	0
2012	0	0	116.86	17.3	415.84	61.6	142.70	21.1	0	0
2013	0	0	97.01	14.4	346.70	51.3	231.69	34.3	0	0
2014	0	0	70.52	10.4	321.87	47.7	283.01	41.9	0	0
2015	0	0	7.47	1.1	254.95	37.7	323.16	47.8	89.72	13.3
2016	0	0	31.38	4.6	104.77	15.5	276.82	41.0	262.52	38.9
2017	0	0	0	0	141.32	20.9	303.22	44.9	240.18	35.6
2018	0	0	0	0	39.69	5.9	241.01	35.7	394.61	58.4

**Table 4:** Landslide activities between 2009 and 2018.

Year	2009	2010	2011	2012	2013	2014	2015	2016	2017	2018
Active (%)	100	99.9	99.9	75.29	65.70	58.09	38.86	20.15	19.55	5.88
Weak active (%)	0	0.01	0.01	24.71	34.30	41.91	61.14	79.85	80.45	94.12

## Data Availability

The data presented in this study are openly available in the landslide inventories: https://www.sciencebase.gov/catalog/item/583f4114e4b04fc80e3c4a1a (accessed on 20 September 2020); the image website: http://www.gscloud.cn/sources/index?pid=2&rootid=2 (accessed on 20 September 2020), https://www.arcgis.com/index.html (accessed on 20 September 2020).

## Supplementary Materials

The following are available online at https://www.mdpi.com/article/10.3390/s21155243/s1, Figure S1: The overall *VDA* map.

## Abbreviation

ASTER	Advanced Spaceborne Thermal Emission and Reflection Radiometer
DEM	Digital Elevation Model
ETM+	(Landsat) Enhanced Thematic Mapper
EVI	Enhanced Vegetation Index
GDEM	Global Digital Elevation Model
HJ-1 T	(Chinese) Huanjing-1 Satellite
MODIS	Moderate-Resolution Imaging Spectroradiometer
MVC	Maximum-Value Composite
*NDVI*	Normalized Difference Vegetation Index
*NIR*	Near-Infrared
OLI	(Landsat 8) Operational Land Imager
SLC	(Landsat 7) Scan Line Corrector
SPOT	(French) Systeme Probatoire d’Observation de la Terre
TIRS	(Landsat 8) Thermal Infrared Sensor
TM	(Landsat) Thematic Mapper
*VDA*	Vegetation Damage
*VRR*	Vegetation Recovery Rate
