# Supplementary material for: Discovering Vegetation Recovery and Landslide Activities in the Wenchuan Earthquake Area with Landsat Imagery
Source: Sensors (Basel). 2021 Aug 3;21(15):5243. doi: 10.3390/s21155243 (PMC8347366; doi:10.3390/s21155243)
Supplement: Supplementary file 1 [file sensors-21-05243-s001.zip › sensors-1294455-supplementary.pdf]

*Article*

# Discovering Vegetation Recovery and Landslide Activities in the Wenchuan Earthquake Area with Landsat Imagery

**Cheng Zhong [1], Chang Li [1], Peng Gao [2,3] and Hui Li [4,*]**

[1] Three Gorges Research Center for Geo-hazard, Ministry of Education, China University of Geosciences, Wuhan 430074, China; zhonglxm@cug.edu.cn (C.Z.); lichang_net@cug.edu.cn (C.L.)

[2] Department of Earth and Ocean Sciences, University of North Carolina, Wilmington, NC 28403, USA; gaop@uncw.edu

[3] Department of Geography, University of South Carolina, Columbia, SC 29208, USA

[4] School of Earth Sciences, China University of Geosciences, Wuhan 430074, China

* Correspondence: rslihui@cug.edu.cn; Tel.: +86-138-0861-8138

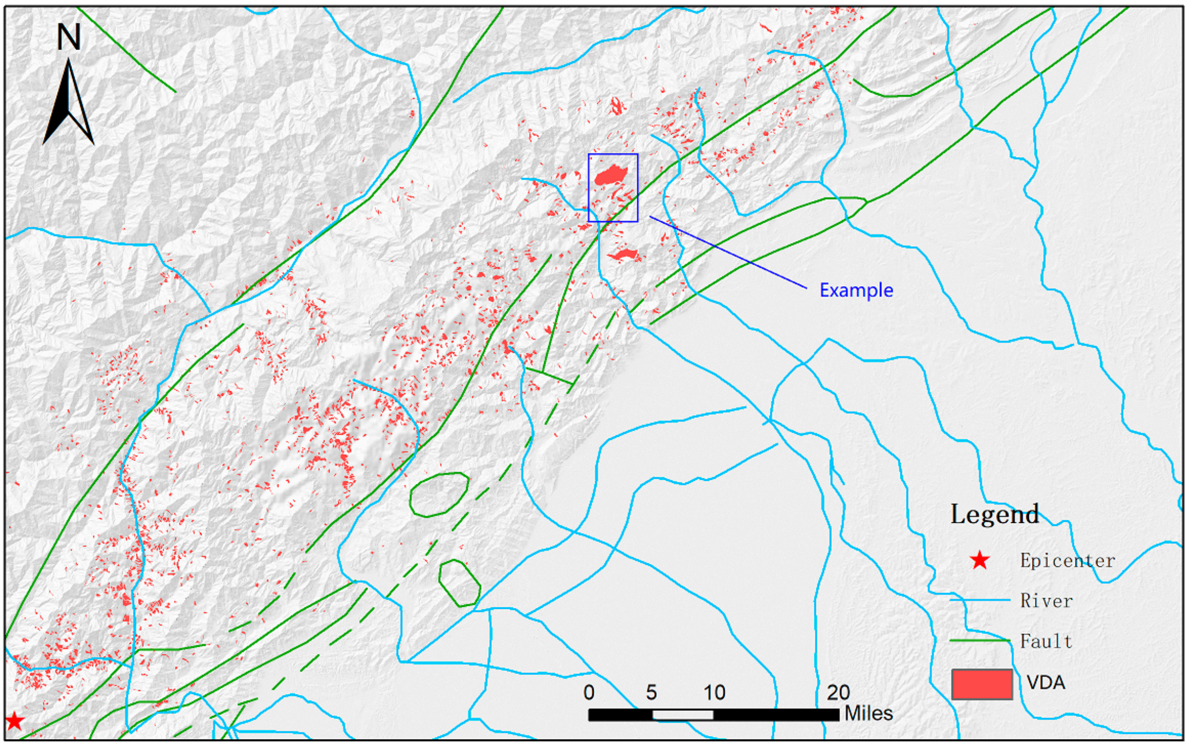


**Figure S1.** The overall VDA map. The VDA map is in accordance with the landslide map, as the latter is visually interpreted from images according to the vegetation damage status.
